# MRI-Based Delta Necrosis as a Prognostic Marker Following Neoadjuvant Chemotherapy in Soft Tissue Sarcoma

**DOI:** 10.3390/cancers18020291

**Published:** 2026-01-17

**Authors:** Harold Bravo Thompson, Priya Chattopadhyay, Ty Subhawong, Malcolm-Christopher Palmer, Sergio Torralbas Fitz, Brooke Crawford, Andrew Rosenberg, H. Thomas Temple, Emily Jonczak

**Affiliations:** 1 Department of Medicine, University of Miami/Jackson Health System, Miami, Fl 33136, USA; pxc804@miami.edu; 2 Department of Radiology, University of Miami Heath System, Miami, FL 33136, USA; tsubhawong@med.miami.edu (T.S.); malcolm.palmer@med.miami.edu (M.-C.P.); 3 Department of Orthopedics, Musculoskeletal Oncology Division, University of Miami Heath System, Miami, FL 33136, USA; sjt96@med.miami.edu (S.T.F.); bxc859@med.miami.edu (B.C.); htemple@med.miami.edu (H.T.T.); 4 Department of Pathology & Laboratory Medicine, University of Miami Heath System, Miami, FL 33136, USA; arosenberg@med.miami.edu; 5 Sylvester Comprehensive Cancer Center, Sarcoma Medical Oncology, University of Miami Heath System, Miami, FL 33136, USA; emily.jonczak@med.miami.edu

**Keywords:** soft tissue sarcoma, necrosis, prognostic factor, neoadjuvant therapy

## Abstract

**Background:** The prognostic value of treatment-induced necrosis in soft STS remains uncertain. This study evaluated whether MRI-based changes in necrosis (Δ necrosis) between pre- and post-neoadjuvant chemotherapy scans correlate with pathologic necrosis and clinical outcomes. **Methods:** In this retrospective cohort, 27 patients with STS who received neoadjuvant chemotherapy and underwent pre- and post-treatment MRI were analyzed. Necrosis was graded categorically (<5%, 5–25%, 25–50%, 50–75%, 75–95%, and >95%), and Δ necrosis was calculated as the change in estimated necrosis between scans. Correlations between MRI-derived and pathologic necrosis were assessed using Spearman’s rank coefficient. Survival analyses (progression-free, local recurrence-free, and disease-specific overall survival) were performed using Kaplan–Meier and log-rank tests. **Results:** Post-treatment MRI necrosis moderately correlated with pathologic necrosis (ρ = 0.44, *p* = 0.028), whereas Δ necrosis showed a weaker, nonsignificant correlation (ρ = 0.24, *p* = 0.24). Neither MRI-based nor pathologic necrosis thresholds were associated with survival outcomes. **Conclusions:** MRI-based Δ necrosis did not predict pathologic necrosis or oncologic outcomes in STS, suggesting that radiologic changes in necrosis may not serve as reliable markers of therapeutic response. Future studies integrating quantitative imaging and standardized pathology protocols together with future exploration of molecular tools such as ctDNA are needed to refine treatment assessment in STS.

## Simple Summary

Soft tissue sarcomas (STS) are rare and diverse tumors for which neoadjuvant chemotherapy is often used to improve local control and survival. However, accurately determining treatment effectiveness remains challenging. This study evaluated whether changes in tumor necrosis measured on MRI scans before and after chemotherapy, referred to as delta necrosis, could predict outcomes or mirror the amount of necrosis seen in surgical pathology. We found that MRI-based delta necrosis did not correlate with pathologic necrosis or patient survival, suggesting it may not serve as a reliable prognostic marker in STS. Standardized imaging and pathology assessment protocols, together with future exploration of molecular tools such as ctDNA, may help refine response evaluation and improve prognostic accuracy in this heterogeneous disease.

## 1. Introduction

Soft tissue sarcomas (STS) are rare and heterogeneous malignant tumors of mesenchymal origin, representing approximately 1% of adult cancers. They show a modest male predominance (1.4:1) and most commonly arise from skeletal muscle, connective tissue, or adipose tissue [1,2].

Despite advances in multimodal therapy, local recurrence occurs in 10–20% of cases and distant metastases in about 30%. Surgery with adjuvant radiotherapy achieves local control rates close to 90%, yet high-grade tumors remain aggressive, with a median survival of roughly 47 months for grade III disease. Prognosis varies by histologic subtype and anatomic site, and treatment-related morbidity can substantially affect quality of life [1,2,3].

Several clinicopathologic variables, including tumor grade, size, depth, location, histologic subtype, and margin status, have been identified as independent prognostic factors [4]. However, the rarity and biological diversity of STS, combined with variations in treatment strategies and response, continue to limit the development of standardized, evidence-based approaches.

Histopathologic tumor viability, defined as the proportion of viable tumor cells remaining after neoadjuvant therapy, has been proposed as a prognostic biomarker across multiple malignancies. In bone sarcomas, residual viability greater than 10% correlates with increased local recurrence and metastasis. Its role in STS, however, remains uncertain, with prior studies reporting conflicting results [2,3,4]. A previous investigation from our institution found no significant association between histopathologic viability and oncologic outcomes, regardless of neoadjuvant therapy or tumor characteristics [2].

Assessment of treatment responses in STS using conventional imaging remains challenging. Due to their heterogeneous internal composition, tumors that respond biologically to chemotherapy may not exhibit size reduction because of treatment-induced changes such as cystic degeneration, fibrosis, necrosis, or hemorrhage. Consequently, size-based criteria such as WHO or RECIST may underestimate therapeutic response in these tumors [5].

Magnetic resonance imaging (MRI) remains the gold standard modality for the evaluation of STS. Established prognostic factors in STS include histologic grade, tumor size, the extent of necrosis, and the presence of metastatic disease at diagnosis. MRI provides an accurate assessment of lesion size and necrotic components, as well as certain imaging characteristics that may correlate with histologic grade [1].

A recent investigation explored the use of a delta-radiomics framework to predict chemotherapeutic response in STS by analyzing temporal variations in MRI-derived imaging features before and after neoadjuvant chemotherapy. However, in contrast to earlier reports, that study did not demonstrate a significant predictive capability for MRI-based delta-radiomics models in assessing treatment response among patients with STS [5].

Building upon this background, the present study investigates MRI-based changes in tumor necrosis, termed delta necrosis, between pre- and post-neoadjuvant chemotherapy using a semi-quantitative, clinically applicable imaging approach. Unlike prior studies focused on static pathological necrosis or high-dimensional radiomics models, this work evaluates longitudinal changes in necrosis assessed through routine MRI interpretation, reflecting real-world clinical practice. By examining the prognostic relevance and limitations of MRI-based Δ necrosis in a heterogeneous STS cohort, this study aims to clarify whether dynamic imaging-derived necrotic change provides incremental clinical information and to define the boundaries of its utility as an exploratory imaging biomarker in soft tissue sarcoma.

## 2. Materials and Methods

This retrospective cohort study was conducted at the same institution as the prior work by Montreuil et al. [2] and was designed as an exploratory analytical imaging investigation evaluating longitudinal, semi-quantitative MRI-based changes in tumor necrosis (Δ necrosis) in patients with soft tissue sarcoma. The source population included 147 patients surgically treated for soft tissue sarcoma between 2010 and 2021. Using this institutional database—subsequently updated to include cases through 2023—patients were screened for receipt of neoadjuvant chemotherapy and availability of MRI scans both before initiation of chemotherapy (MRI-1) and after completion of chemotherapy but prior to surgical resection (MRI-2).

**Figure 1:**
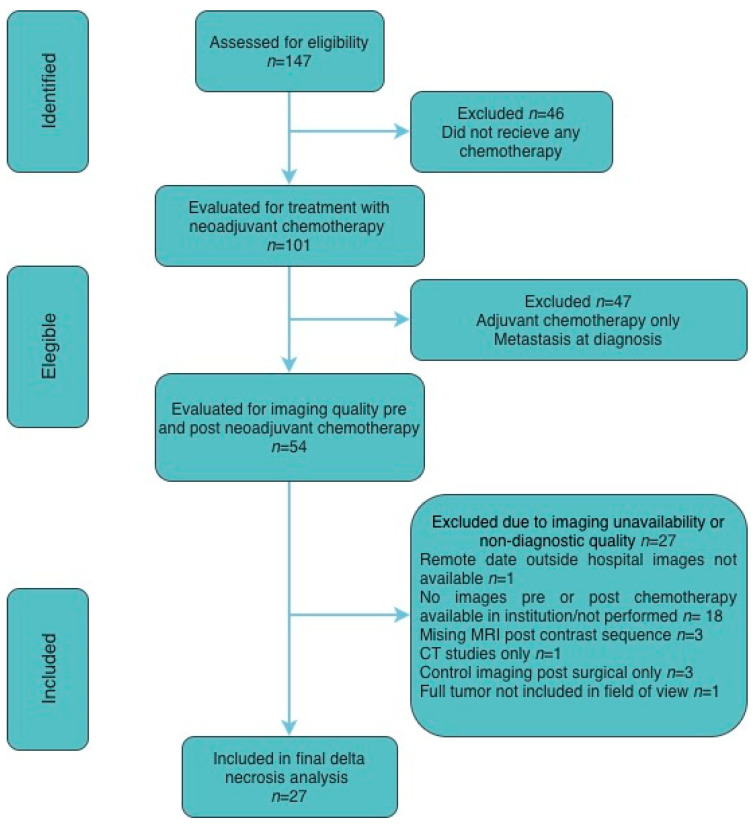
CONSORT-style flow diagram illustrating patient selection and exclusion criteria. A total of 147 patients were assessed for eligibility. Sequential exclusions were applied based on receipt of neoadjuvant chemotherapy, clinical eligibility, and availability of diagnostic-quality pre- and post-treatment imaging. Patients were excluded for predefined clinical reasons (absence of neoadjuvant chemotherapy, adjuvant-only treatment, or metastatic disease at diagnosis) or technical imaging limitations (unavailable, incomplete, or non-diagnostic imaging studies). The final analytic cohort comprised 27 patients included in the delta necrosis analysis.

Patients were identified retrospectively and sequentially screened for eligibility, receipt of neoadjuvant chemotherapy, and availability of diagnostic-quality pre- and post-treatment imaging. Exclusions were based on predefined clinical criteria (absence of neoadjuvant chemotherapy, metastatic disease at diagnosis, or adjuvant-only treatment) and technical imaging limitations (missing or non-diagnostic MRI sequences or unavailable studies), resulting in a final cohort of 27 patients included in the analysis. A CONSORT-style diagram is included for details (Figure 1).

Demographic and clinical data were collected for each patient, including age, sex, and receipt of preoperative or postoperative chemotherapy. MRI examinations were reviewed by an experienced musculoskeletal radiologist, who assigned tumor necrosis for both baseline (MRI-1) and post-treatment (MRI-2) studies using the following semi-quantitative categories: <5%, 5–25%, 25–50%, 50–75%, 75–95%, and >95%. Given the known overlap between necrotic tissue and post-treatment cystic or fibrotic changes on MRI, this semi-quantitative approach using broad necrosis categories was intentionally employed to reduce misclassification bias and interobserver variability, and to reflect routine clinical practice.

Because necrosis was recorded as categorical ranges, delta necrosis does not follow the same distribution pattern, as its values are generally smaller in magnitude and may be either positive or negative. Thus, delta necrosis was calculated by assuming a median value for each category to estimate the percentage change between MRI 1 and MRI 2.

In this retrospective cohort, pathological sampling followed routine institutional practice at the time of resection, with extensive tumor sampling across the maximal tumor dimension, including one to two complete cross-sectional slabs and additional sampling of the remaining tumor, resulting in submission of the entire tumor for histologic review, with an average of approximately two sections per centimeter of tumor. The extent of tumor necrosis was estimated for each specimen and expressed as a total percentage, as determined by a single sarcoma pathologist. Clinical outcomes, including local recurrence, distant metastasis, treatment-related complications, and mortality, were abstracted from follow-up records documented by the primary orthopedic oncology surgeon and sarcoma oncologist.

Quantitative variables were expressed as mean ± standard deviation, depending on data distribution. Categorical variables were summarized as counts and percentages. Group comparisons were performed using chi-square or Fisher’s exact tests for categorical data and Student’s *t*-test or Mann–Whitney U test for continuous data, as appropriate.

To evaluate the association between MRI-based and pathological necrosis, correlation analyses were performed using data from patients with available pre- and post-chemotherapy MRI studies and corresponding surgical pathology specimens. Correlations between MRI-derived necrosis and pathological necrosis were assessed using Spearman’s rank correlation coefficient (ρ) due to the non-parametric distribution of the data. Two MRI-derived variables were analyzed: (1) the absolute necrosis percentage measured on post-chemotherapy MRI (Scan 2) and (2) the change in necrosis between baseline and post-chemotherapy MRI (Δ necrosis, expressed in percentage points).

Receiver operating characteristic (ROC) curve analysis was conducted to evaluate the ability of these MRI parameters to discriminate against cases with high pathological necrosis (≥80%). The optimal cutoff for each MRI-derived variable was determined from the ROC analysis, identifying ≥6.5 percentage points for Δ necrosis and the corresponding threshold for post-chemotherapy MRI necrosis. However, since a 6% difference may not be clinically meaningful, the authors also explored thresholds of 20% and 50 to assess potential clinical relevance.

Diagnostic performance at these thresholds was evaluated by calculating sensitivity, specificity, overall accuracy, positive predictive value (PPV), and negative predictive value (NPV) using 2 × 2 contingency tables comparing MRI-based and pathological classifications. Agreement between MRI-derived and pathological necrosis categories was assessed using Cohen’s kappa (κ) statistic. Statistical significance for κ was determined using the Z-test.

Survival outcomes were analyzed to explore the prognostic relevance of necrosis assessed by pathology and MRI-derived metrics. Progression-free survival (PFS), local recurrence-free survival (LRFS), and disease-specific overall survival (OS) were evaluated using the Kaplan–Meier method, with group comparisons performed using the log-rank test.

Progression was defined according to RECIST 1.1 criteria as an increase of at least 20% in tumor size compared with the smallest recorded measurement during follow-up. Local recurrence was defined as clinically confirmed disease recurrence within the primary tumor bed or surgical site, as determined by the treating oncologist during post-surgical surveillance, and represented the true local progression of disease after initial surgical control. Disease-specific overall survival was defined as the time from diagnosis to death attributable to sarcoma, with deaths unrelated to disease or treatment censored at the last follow-up.

For all outcomes, patients without an event (progression, recurrence, or disease-related death) at last contact were censored at the date of their most recent clinical or imaging assessment. Survival endpoints were compared across necrosis categories defined by pathology, post-chemotherapy MRI necrosis, and Δ necrosis thresholds of ≥6.5, ≥20, and ≥50 percentage points.

All analyses were performed using STATA version 18.0 (StataCorp, College Station, TX, USA) and R version 3.5.1 (R Foundation for Statistical Computing, Vienna, Austria). Statistical significance was defined as *p* < 0.05. Ethical approval for this study was obtained from the University of Miami Institutional Review Board (IRB #20230560).

## 3. Results

**Figure 2:**
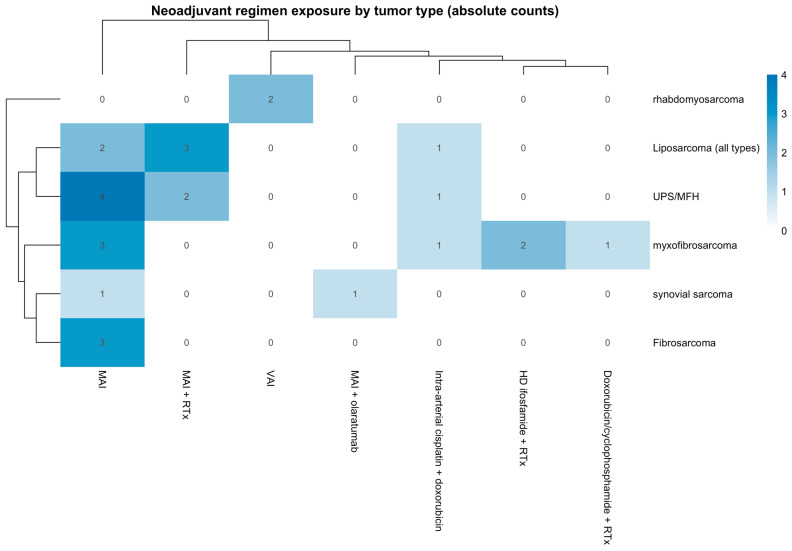
Heatmap evaluation of the distribution of neoadjuvant chemotherapy regimens across tumor histologies. Cells indicate the absolute number of patients with each tumor type exposed to the corresponding regimen; counts are non–mutually exclusive. Regimens include MAI (mesna, doxorubicin, and ifosfamide), MAI with radiotherapy (MAI + RTx), MAI with olaratumab, intra-arterial cisplatin with doxorubicin, high-dose ifosfamide with radiotherapy, doxorubicin with cyclophosphamide and radiotherapy, and VAI (vincristine, dactinomycin, and ifosfamide).

A total of 27 patients with STS were included for analysis in this study. The study included 69.7% (18) males and 33.3% (9) females, with a variety of histological tumor types, including 22.2% (6) liposarcoma [all types including myxoid and pleomorphic liposarcoma], 7.4% (2) synovial sarcoma, 25.95% (7) undifferentiated pleomorphic sarcoma/malignant fibrous histiocytoma (UPS/MFH), 11.1% (3) fibrosarcoma, 25.95% (7) myxofibrosarcoma, and 7.4% (2) rhabdomyosarcoma. The distribution of neoadjuvant chemotherapy regimens across tumor histologies is summarized in Figure 2, illustrating treatment heterogeneity within the cohort. Regimens are reported at the regimen level without cycle counts to preserve patient confidentiality in this single-institution rare-tumor cohort.

Additional pathologic and imaging characteristics, including baseline and post-chemotherapy necrosis ranges, the mean volume of mass, and the largest diameter of mass, among others, are presented in Table 1.

In this cohort, 5 (18.5%) patients presented with local recurrences during follow-up, and 3 (11.1) patients presented metastatic disease. The mean time to recurrence was 615.8 days ± 245.5 days. Additional prognostic baseline characteristics are presented in Table 1.

**Figure 3:**
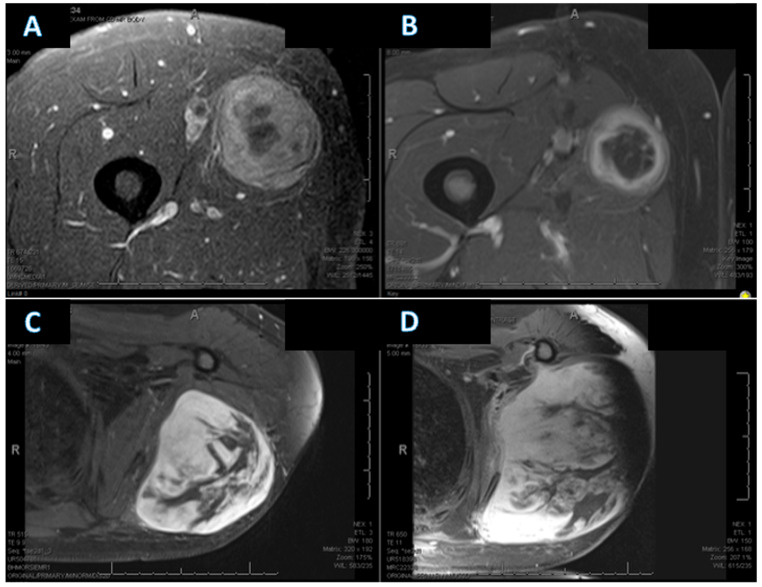
Representative MRI examples illustrating evaluation of changes in tumor necrosis following neoadjuvant chemotherapy: (**A**,**B**) Example demonstrating a substantial increase in necrosis (Δ necrosis 47.5%). (**A**) Baseline MRI (MRI-1) showing an estimated necrosis of 25–50%. (**B**) Post-neoadjuvant MRI (MRI-2) demonstrating increased necrosis, estimated at 75–95%. (**C**,**D**) Example demonstrating no change in necrosis (Δ necrosis 0%). (**C**) Baseline MRI (MRI-1) with estimated necrosis of 25–50%. (**D**) Post-neoadjuvant MRI (MRI-2) with unchanged estimated necrosis (25–50%). All images were obtained from the electronic medical record, and representative axial slices were selected by a dedicated musculoskeletal radiologist.

Representative pre- and post-treatment MRI examples illustrating changes in tumor necrosis are shown in Figure 3, including one case with a marked increase in necrosis (Δ necrosis 47.5%) and one case with no change in necrosis (Δ necrosis 0%).

To evaluate the clinical and pathological relevance of MRI-based Δ necrosis, multiple complementary analyses were performed, including correlation with histopathologic necrosis and threshold-based survival analyses.

### 3.1. Diagnostic Accuracy

A correlation analysis was performed to evaluate the relationship between MRI-based and pathological necrosis. The change in necrosis between pre- and post-chemotherapy MRI (Δ necrosis) demonstrated a weak, nonsignificant correlation with pathological necrosis (Spearman’s ρ = 0.24, *p* = 0.238). In contrast, necrosis measured on post-chemotherapy MRI showed a moderate, statistically significant correlation with pathological necrosis (Spearman’s ρ = 0.44, *p* = 0.0279).

Receiver operating characteristic (ROC) analysis was performed to evaluate the ability of MRI-derived necrosis metrics to discriminate against cases with high pathological necrosis. The necrosis percentage measured on post-chemotherapy MRI demonstrated good diagnostic performance, with an area under the curve (AUC) of 0.769, indicating acceptable accuracy in predicting pathological necrosis. In contrast, Δ necrosis showed only modest discriminative ability, with an AUC of 0.617. These findings support that the absolute percentage of necrosis observed on post-treatment MRI better reflects the extent of pathological necrosis than the relative change between scans.

In the main delta necrosis analysis, the authors dichotomized the variable using the optimal cutoff point derived from the ROC curve (6.25 percentage points). However, since a 6% difference may not be clinically meaningful, the authors also explored thresholds of 20% and 50% to assess potential clinical relevance. An exploratory analysis of different Δ necrosis thresholds was performed to assess their impact on diagnostic classification relative to pathological necrosis ≥ 80%. Using a cutoff of Δ necrosis ≥ 6.5 percentage points, MRI achieved high sensitivity (0.91) but low specificity (0.21), correctly identifying most patients with high pathological necrosis while overestimating responders among those with poor histologic response. Increasing the threshold to ≥20 pp resulted in comparable overall accuracy but slightly reduced sensitivity, whereas a stricter cutoff of ≥50 pp markedly decreased sensitivity with minimal gain in specificity. These results indicate that lower Δ necrosis thresholds improve sensitivity but compromise specificity, supporting the ROC-derived cutoff of 6.5 pp as the most balanced criterion for identifying patients with high necrosis on pathology.

Using the optimal ROC-derived cutoff, necrosis measured on post-chemotherapy MRI (Scan 2) achieved a sensitivity of 0.91, specificity 0.50, and overall accuracy 0.68, with a positive predictive value (PPV) of 0.59 and negative predictive value (NPV) of 0.88. In comparison, Δ necrosis ≥ 6.5 pp showed lower sensitivity (0.36) but higher specificity (0.86), resulting in a comparable overall accuracy of 0.64 (PPV 0.67, NPV 0.63). Agreement analysis using Cohen’s κ demonstrated moderate concordance for post-chemotherapy MRI necrosis (κ = 0.387, Z = 2.18, *p* = 0.030) and fair concordance for Δ necrosis (κ = 0.232, Z = 1.28, *p* = 0.199).

### 3.2. Survival Analysis

To determine whether necrosis assessed by MRI or pathology was associated with patient outcomes, survival analyses were conducted evaluating progression-free survival (PFS), local recurrence-free survival (LRFS), and disease-specific overall survival (OS) according to pathological and MRI-based necrosis categories.

#### 3.2.1. Progression-Free Survival

**Figure 4:**
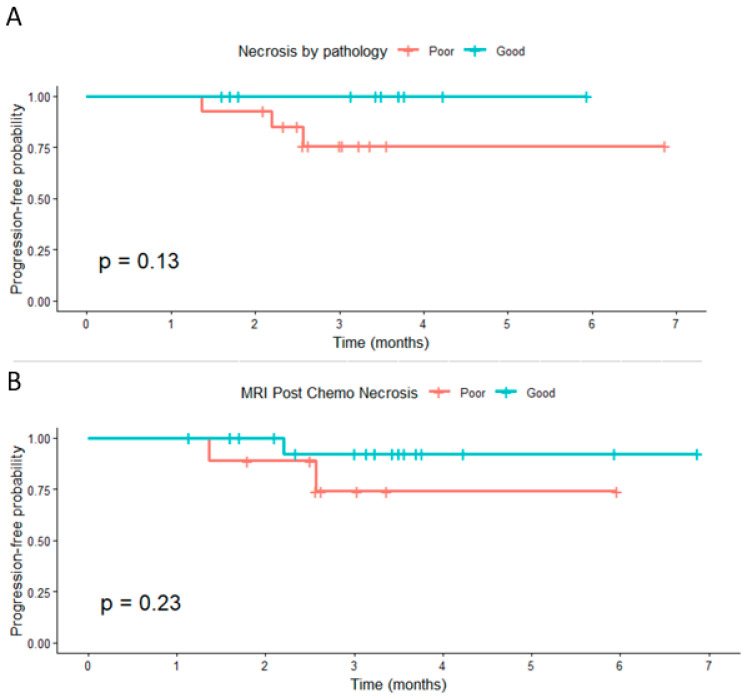
Progression-free survival by pathologic and MRI-based necrosis: (**A**) Kaplan–Meier curve showing progression-free survival (PFS) according to pathologic necrosis (≥80% vs. <80%). Patients with higher necrosis demonstrated a trend toward improved PFS (*p* = 0.13). (**B**) Kaplan–Meier curve showing PFS according to post-chemotherapy MRI necrosis categorized as good or poor response. A similar nonsignificant trend was observed (*p* = 0.23).

Patients with high pathological necrosis (≥80%) showed a nonsignificant trend toward improved PFS compared with those with poor necrosis (*p* = 0.13). Similarly, post-chemotherapy MRI necrosis was not significantly associated with PFS (*p* = 0.23) (Figure 4). When categorized by Δ necrosis thresholds, no significant differences in PFS were observed for ≥6.5 pp, ≥20 pp, or ≥50 pp (*p* = 0.83, 0.92, and 0.18, respectively). Despite the lack of statistical significance, patients classified as good responders generally demonstrated slightly higher short-term progression-free probabilities.

Censoring marks represent patients without progression at the last follow-up. Progression was defined as an increase of ≥20% in tumor size per RECIST 1.1.

#### 3.2.2. Local Recurrence-Free Survival

For LRFS, no significant associations were identified across Δ necrosis thresholds of ≥6.5 pp, ≥20 pp, or ≥50 pp (*p* = 0.64, 0.52, and 0.73, respectively). Although good responders tended to maintain higher recurrence-free probabilities over time, survival curves overlapped substantially, and event numbers were low.

#### 3.2.3. Disease-Specific Overall Survival

**Figure 5:**
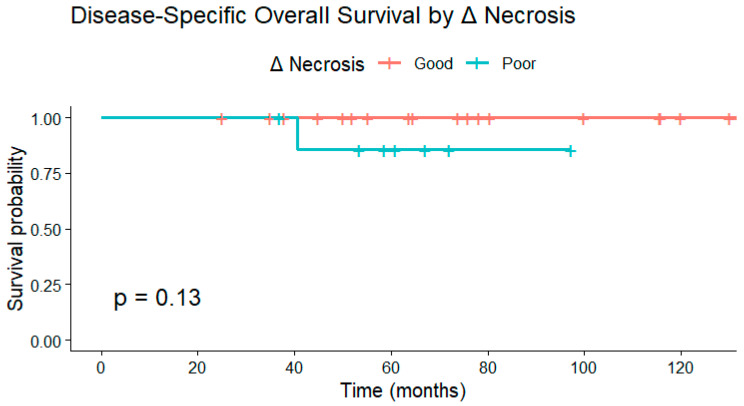
Disease-specific overall survival by change in necrosis (Δ necrosis ≥ 6.5 percentage points).

Similarly, disease-specific overall survival did not differ significantly between necrosis response groups at any Δ necrosis cutoff. Kaplan–Meier analyses demonstrated *p* = 0.13 for ≥6.5 pp, *p* = 0.17 for ≥20 pp, and *p* = 0.65 for ≥50 pp. Across thresholds, survival probabilities remained high in both groups, reflecting limited mortality within the follow-up period (Figure 5).

Overall, while patients exhibiting higher necrosis, whether determined pathologically or radiologically, tended to experience numerically better outcomes, none of the imaging-based or Δ necrosis thresholds reached statistical significance for progression-free, local recurrence-free, or disease-specific overall survival. Radiologic Δ necrosis was not significantly associated with recurrence-free or overall survival, outcomes known to be influenced by surgical and histopathologic factors.

Kaplan–Meier curve demonstrating disease-specific overall survival (OS) stratified by Δ necrosis response. Although patients with greater increases in necrosis tended to have higher survival probabilities, no statistically significant difference was observed (*p* = 0.13). Deaths unrelated to sarcoma were censored at the date of the last follow-up.

## 4. Discussion

In this study, MRI-based changes in necrosis (Δ necrosis) before and after neoadjuvant chemotherapy did not significantly correlate with pathological necrosis nor predict progression-free, local recurrence-free, or disease-specific overall survival. Although post-chemotherapy MRI necrosis demonstrated a moderate correlation with histopathologic necrosis, the relative change between pre- and post-treatment imaging showed limited predictive value. These findings suggest that, in soft tissue sarcoma (STS), the extent of necrosis assessed radiologically or histologically may not independently reflect treatment efficacy or long-term outcomes, consistent with prior evidence questioning the prognostic role of necrosis in this setting [2,4,6,7]. Importantly, unlike prior institutional studies that evaluated necrosis as a static post-resection parameter, the present work examines longitudinal radiologic Δ necrosis on MRI before and after neoadjuvant therapy, providing a dynamic, non-invasive assessment that reflects real-world clinical practice.

Montreuil et al. reported that patients with ≥90% necrosis at resection did not experience improved local control, metastasis-free survival, or overall survival compared with those with lower degrees of necrosis. A considerable proportion of tumors that did not receive neoadjuvant therapy also exhibited spontaneous necrosis, consistent with observations by Mullen et al. and Menendez et al., who found no survival advantage associated with greater necrosis [2,4,6,7]. Treatment-induced necrosis is a well-established prognostic marker in malignant bone tumors [4,7], but its predictive value in STS remains uncertain. The extent of necrosis following therapy may reflect both tumor chemosensitivity and intrinsic biological behavior. However, even in a relatively large cohort of 82 patients, Menendez et al. failed to identify a survival difference based on necrosis percentage [4].

Tumor necrosis may result from chemotherapy or arise spontaneously. Prior studies have shown that up to 40% of necrosis in STS can occur independently of treatment [4,8], and similar rates have been described in osteosarcoma managed with surgery alone [4,7]. In osteogenic sarcoma, necrosis ≥95% is considered a marker of excellent histologic response and improved prognosis [7,9], yet Menendez et al. found no survival difference between STS patients with ≥95% necrosis and those with lesser degrees [4]. This contrast reinforces that necrosis as a prognostic indicator may differ fundamentally between bone and soft tissue sarcomas.

While several studies have reported correlations between necrosis and survival in STS, findings remain inconsistent. MacDermed et al. identified a significant association between necrosis >90% and improved distant metastasis-free survival (*p* = 0.029) [9], and Monsky et al. [10] demonstrated that semi-automated volumetric MRI segmentation allows for more accurate quantification of necrosis throughout the tumor volume. Conversely, Gannon et al. observed a paradoxical association between higher necrosis and poorer survival outcomes, and Fromm et al. found no association between necrosis and overall or disease-free survival in STS, despite confirming its predictive value in bone sarcomas [11,12,13]. Collectively, these contradictory findings, together with our results, support a heterogeneity-based model of STS, in which necrosis, whether assessed radiologically or histologically, more likely reflects intrinsic tumor biology and microenvironmental factors rather than the direct efficacy of neoadjuvant chemotherapy [14,15]. In this context, disease-specific survival in our cohort should be interpreted cautiously, as it was likely influenced by heterogeneity in systemic therapies and variability in the interval between baseline and post-treatment MRI, including the potential administration of subsequent lines of therapy; accordingly, survival outcomes were included for descriptive purposes and were not intended to reflect the efficacy of specific chemotherapy regimens.

Quantitative imaging approaches such as radiomics have been proposed as tools to improve response assessment. Radiomic features extracted from pre-treatment imaging have shown potential in predicting survival, progression, and tumor grade in STS [16,17,18]. Crombé et al. achieved an AUC of 0.63 using T2-weighted delta-radiomics to predict pathologic complete response after chemotherapy [16,19], while Lin et al. reported an AUC of 0.82 in osteosarcoma using CT-based delta-radiomics [20]. Similarly, Peeken et al. observed AUC values of 0.68–0.75 in high-grade STS [16]. Unlike radiomics-based approaches, which rely on high-dimensional quantitative feature extraction, the present study employed a semi-quantitative assessment of necrosis derived from routine MRI interpretation, reflecting real-world clinical practice and aiming to evaluate the translational applicability of Δ necrosis as an exploratory imaging marker. However, our results indicate that simple categorical assessments of MRI-based necrosis, while clinically accessible, may not capture the nuanced treatment-related changes that quantitative radiomic models can identify. When evaluated against established benchmarks, including histopathologic necrosis and survival-based outcomes, MRI-based Δ necrosis did not demonstrate superior prognostic performance in this heterogeneous STS cohort.

Challenges also persist in imaging-based response evaluation. Fadli et al. demonstrated that half of STS exhibited an apparent size increase of >19.8%, which would qualify as progressive disease under RECIST criteria [21,22]. Without re-baselining MRI after neoadjuvant therapy, more than 50% of tumors could have been misclassified as progressing despite treatment efficacy. In addition, approximately one-quarter of patients showed necrotic signal changes before therapy initiation, which could confound response assessment using enhancement-based criteria such as Choi or modified Choi [22,23,24,25,26,27]. These findings underscore the importance of integrating imaging and pathology for a more comprehensive interpretation of response dynamics. Emerging molecular tools such as circulating tumor DNA (ctDNA) also warrant evaluation as potential prognostic and response biomarkers, as they may complement imaging-based criteria by providing noninvasive molecular insights into tumor burden and treatment efficacy. However, their clinical application in STS remains limited by low cfDNA shedding and subtype heterogeneity, necessitating further methodological refinement and validation [28,29].

Insights from recent Sarcoma Academy consensus discussions further emphasize that necrosis in STS results from a complex interplay of intrinsic tumor biology and treatment effects, producing variable and sometimes conflicting prognostic associations [30]. Therapy-induced necrosis alone cannot serve as a definitive prognostic marker, as high necrosis may reflect either aggressive biology or an effective cytotoxic response.

Differentiating spontaneous necrosis (as defined in FNCLCC grading) from treatment-related cell death remains challenging. Standardized pathological assessment focusing on viable tumor-cell percentage, rather than total necrosis, may provide greater consistency across centers. Harmonized pathology–imaging protocols, including uniform thresholds and standardized response criteria such as RECIST 1.1 or PERCIST, are critical to improving reproducibility in future multicenter studies [24,30,31].

Limitations of the present study include the small sample size, which restricts statistical power, and its retrospective design, which may introduce selection bias. Necrosis was measured categorically rather than using continuous quantitative metrics, and dichotomization of Δ necrosis thresholds may have reduced sensitivity. The assumption of median values within categorical ranges introduces additional uncertainty. In addition, given the semi-quantitative design and limited cohort size, intratumoral heterogeneity and subtype-specific necrotic patterns were not formally assessed and warrant evaluation in future radiomics-based studies. Furthermore, the limited number of survival events precluded adequate statistical power to detect survival differences; therefore, survival analyses were included and interpreted in an exploratory manner. Despite these limitations, this study contributes to the growing body of evidence indicating that MRI-based Δ necrosis alone may have limited prognostic utility in STS and underscores the need for standardized, quantitative imaging and pathologic evaluation frameworks to more accurately characterize treatment response.

## 5. Conclusions

MRI-based Δ necrosis did not correlate with pathologic necrosis or predict clinical outcomes in patients with soft tissue sarcoma treated with neoadjuvant chemotherapy. While post-treatment MRI necrosis demonstrated a moderate association with histopathologic findings, the relative change between scans lacked prognostic significance for progression-free, local recurrence-free, or disease-specific overall survival. By systematically evaluating MRI-based Δ necrosis across multiple analytical frameworks, including correlation with pathology, threshold-based analyses, and survival outcomes, this study provides a clarifying negative result that defines the limitations of Δ necrosis as a standalone prognostic marker in heterogeneous STS. These findings suggest that necrosis, whether assessed radiologically or pathologically, may not independently reflect therapeutic efficacy in STS. However, the potential utility of Δ necrosis in selected subgroups cannot be excluded and should be explored in future studies with larger, stratified cohorts.

Given the biological heterogeneity of soft tissue sarcomas and the multifactorial nature of treatment response, integrating standardized imaging and pathological response criteria will be essential for improving prognostic accuracy. Future multicenter studies should not only employ quantitative imaging and harmonized pathology protocols but also explore emerging molecular tools such as circulating tumor DNA (ctDNA) as complementary biomarkers of treatment response and disease progression, to better define robust predictors that can guide neoadjuvant treatment decisions without delaying surgical management.

## Figures and Tables

**Table 1:** Baseline pathologic, imaging, and prognostic characteristics of the cohort.

Characteristic	N ^1^
MRI 1 (Baseline) Percent Necrosis	27
<5%	3 (11.1%)
5–25%	12 (44.4%)
25–50%	7 (26.0%)
50–75%	3 (11.1%)
75–95%	1 (3.7%)
>95%	1 (3.7%)
Volume of Mass at MRI 1 (cm^3^)	325 (273)
Largest Diameter at MRI 1 (cm)	10.46 (2.95)
MRI 2 (Completion of Chemotherapy) Percent Necrosis	27
<5%	2 (7.4%)
5–25%	3 (11.1%)
25–50%	7 (26.0%)
50–75%	6 (22.2%)
75–95%	9 (33.3%)
Volume of Mass at MRI 2 (cm^3^)	318 (415)
Largest Diameter at MRI 2 (cm)	9.7 (3.6)
Δ Necrosis	24.0 (17.4)
Necrosis by Pathology (%)	70 (31.2)
Δ Volume (cm^3^)	−13.4 (329.4)
Δ Largest Diameter (cm)	−0.7 (3.0)
Δ Volume (%)	−7.4 (83.4)
Δ Largest Diameter (%)	−6.2 (26.8)
Time between Scans (Months)	7.8 (25.9)
Scan 2 Necrosis (Category-see footnote)	27
Good Response	9 (33.3)
Poor Response	18 (67.7)
Necrosis by Pathology (Category)	25
Good Response	14 (56)
Poor Response	11 (44)
Time to recurrence (Days)	615.8 (245.5)
Status	27
NED	19 (70.4)
Active with disease	2 (7.4)
Deceased from disease	1 (3.7)
Deceased from other causes	5 (18.5)

^1^ 1n (%); mean (SD). Progression was defined as an increase of at least 20% in tumor size, according to RECIST 1.1. The cutoff point to categorize MRI 2 necrosis was 80%, and Δ necrosis was 6.5 percentage points. This was obtained from the corresponding ROC curves (see the Results section below). Volume-related variables are reported as mean ± standard deviation for descriptive purposes only and reflect substantial inter-patient heterogeneity.

## Data Availability

The data presented in this study are available upon reasonable request from the corresponding author. The data are not publicly available due to institutional privacy and ethical restrictions.
